# Duplicated gallbladder with stones: Rare anomaly of the biliary system

**DOI:** 10.1016/j.ijscr.2024.110106

**Published:** 2024-07-31

**Authors:** Tsion Ketema Robele, Simeon Mulugeta, Goytom Knfe, Dagmawi Dagne, Enku Shiferaw

**Affiliations:** a St. Paul's hospital millennium medical college, Department of Surgery, Swaziland Street, PO box 1247, Addis Ababa, Ethiopia; b College of Health Sciences, Addis Ababa university, Department of Surgery, Ethiopia; c St. Paul's hospital millennium medical college, Department of Surgery, Ethiopia

**Keywords:** Double gallbladder, Anomalous gallbladder, Open cholecystectomy, Case report

## Abstract

**Introduction and importance:**

Duplication of the gallbladder is a rare congenital anomaly of the biliary system and is associated with an increased risk of complications such as bile duct injury during cholecystectomy.

**Case presentation:**

A 40-year-old woman was admitted for symptomatic cholelithiasis. All preoperative workups were normal, except for an abdominal ultrasound which reported of gallstones. However, during the open cholecystectomy, duplicated gallbladder was an intraoperative surprise. Both gallbladders were successfully removed, and the patient had a smooth recovery without any complications.

**Clinical discussion:**

The presence of a duplicated gallbladder necessitates careful consideration of the biliary ductal and arterial anatomy anomaly to prevent complications during cholecystectomy. While ultrasound is typically used as an initial diagnostic tool for suspected duplicated gallbladder, it can miss the diagnosis of duplicated gallbladder. Laparoscopic cholecystectomy is the preferred method of treatment in an ideal surgical setting.

**Conclusion:**

Duplication of the gallbladder requires special attention to the biliary ductal and arterial anatomy. Preoperative imaging should be helpful for diagnosis.

## Highlights

• Duplicated gallbladder, a congenital malformation characterized by the presence of two distinct gallbladders, is an exceptionally uncommon aberration of the biliary system
• There are no specific symptoms or signs associated with duplicate gallbladders
• Abdominal ultrasound (US) is often the first-line imaging modality used in the assessment of a patient with gallbladder disease; however, it does not always allow a precise diagnosis of gallbladder malformations.
• The presence of duplicated gallbladder poses unique challenges during surgical interventions such as cholecystectomy, as it is associated with an increased risk of complications, including bile duct injury
• Laparoscopic cholecystectomy is the mainstay of treatment in an ideal setup.

## 1 Introduction

Duplicated gallbladder, a congenital malformation characterized by the presence of two distinct gallbladders, is an exceptionally uncommon aberration of the biliary system [1]. The reported incidence of this condition ranges from 1 in 3800 to 4000 cases [2]. The presence of duplicated gallbladder poses unique challenges during surgical interventions such as cholecystectomy, as it is associated with an increased risk of complications, including bile duct injury [3].

Understanding the anatomical variations and potential implications of this rare anomaly is crucial in the diagnosis and management of gallbladder disorders [4]. In this case report, we present a unique case of a duplicated gallbladder with the presence of stones, highlighting its rarity and the significance of appropriate surgical management.

This case report is written following the SCARE criteria [5].

## 2 Case description

A 40-year-old female patient presented with colicky right upper quadrant pain that radiated to the back and worsened after consuming fatty meals. She experienced accompanying symptoms of nausea and intermittent vomiting. She had no pertinent medical history. Physical examination of the abdomen yielded no remarkable findings.

**Fig. 1:**
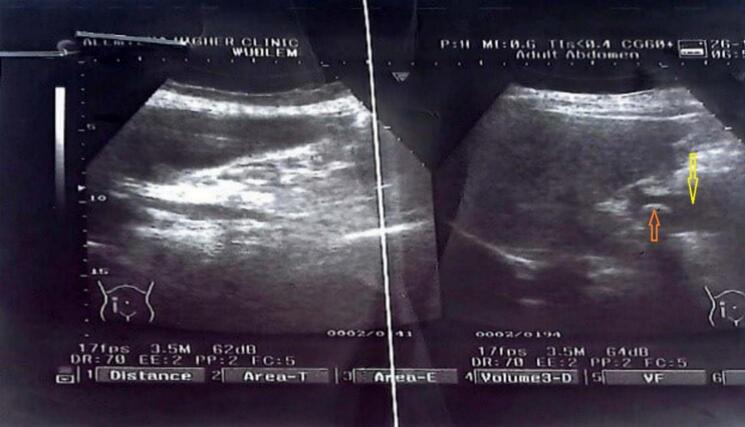
Preoperative ultrasound of a 40 years old female patient with a duplicated gallbladder. Yellow arrow showing anechoic structure indicating the gallbladder and Red arrow showing hyperechoic structure indicating the stone in the gallbladder. (For interpretation of the references to colour in this figure legend, the reader is referred to the web version of this article.)

Laboratory investigations, including complete blood count, liver function tests, renal function tests, serum electrolyte levels, and bilirubin levels, all were within the normal range. An abdominal ultrasound revealed a moderately distended gallbladder with multiple echogenic foci inside the neck and cystic duct, with the largest measuring 1.76 cm in its long axis. Other aspects of the biliary tree and gallbladder wall thickness appeared normal {Fig. 1}. Based on the diagnosis of symptomatic cholelithiasis, the patient was admitted for cholecystectomy.

**Fig. 2:**
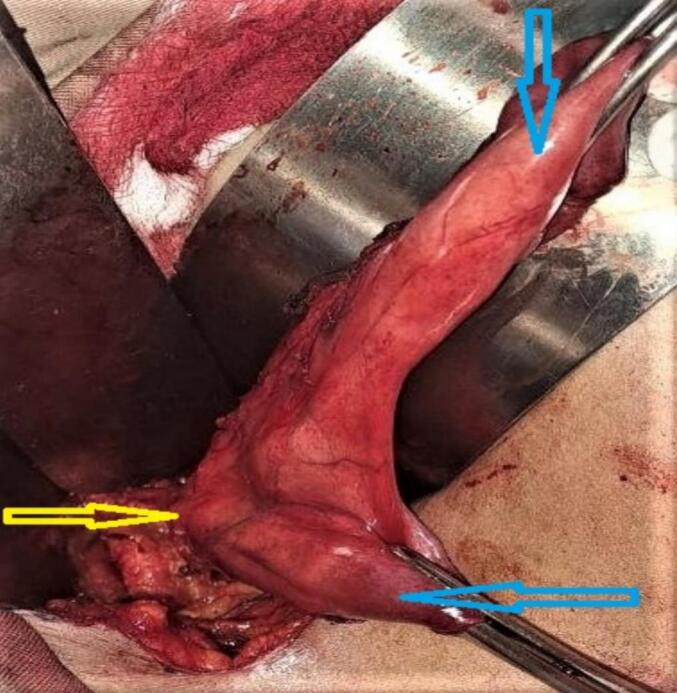
Duplicated gall bladder after dissection showing two clamped gall bladder fundi (blue arrows) and a single cystic duct (yellow arrow). (For interpretation of the references to colour in this figure legend, the reader is referred to the web version of this article.)

**Fig. 3:**
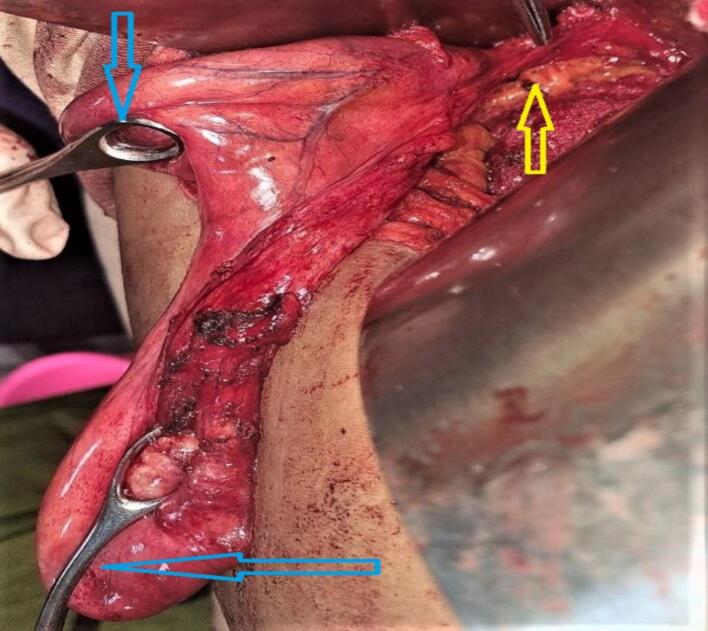
Duplicated gall bladder after dissection showing gall bladder fundi (blue arrows) and a single cystic duct (yellow arrow). (For interpretation of the references to colour in this figure legend, the reader is referred to the web version of this article.)

**Fig. 4:**
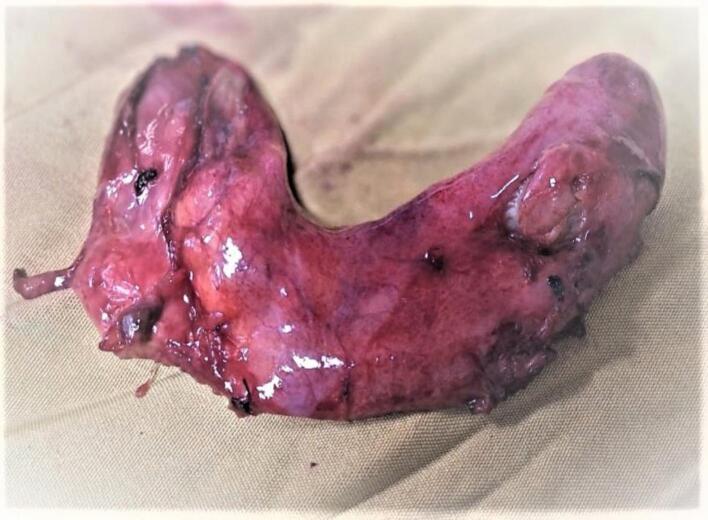
Duplicated gallbladder after removal.

**Fig. 5:**
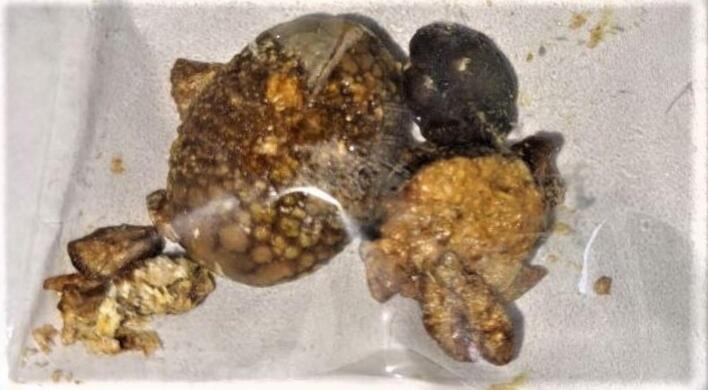
Stones after removal of the duplicated gallbladder.

During the surgical procedure, duplicated gallbladder was unexpectedly encountered, as it had not been detected on the preoperative abdominal ultrasound. Both gallbladders were individually lined by their peritoneal lining, with the lower gallbladder adhered to the duodenum and the upper gallbladder adhered to the liver bed. Following careful dissection and adhesion release, it was observed that the two gallbladders fused at the infundibulum and shared a common cystic duct, which joined the common bile duct. Both gallbladders contained multiple stones {Fig. 2, Fig. 3, Fig. 4, Fig. 5}. Cholecystectomy was successfully performed without complications. The patient had an uneventful postoperative recovery and was discharged on the third day after the surgery. The patient is doing well and improved on the follow up visits.

## 3 Discussion

Duplication of the gallbladder is a rare congenital anomaly, **with an estimated** incidence of one in 4000 live births [1]. Surgeons must be aware of this congenital defect due to the related anatomical differences of the cystic duct and hepatic artery [2]. Congenital malformations of the gallbladder have been categorized into morphological and positional abnormalities including malformation, deformation, multiple gallbladders, ectopias, intrahepatic position, and heterotopic mucosa. Gallbladder duplication is a morphological abnormality [4].

It is considered to result from the incorrect differentiation or excessive division of embryonic organs during the 5th and 6th gestational week when the caudal bud of the hepatic diverticulum splits into separate buds or outpouchings. The later the single primordium bifurcates, the less complete the resulting duplication of the gallbladder. As a result, a true duplication of gallbladder takes place earlier in the gestation and involves the existence of an accessory gallbladder and two distinct cystic ducts. The first reported human case was noted in a sacrificial victim of Emperor Augustus in 31 BCE. Sherren reported the first documented case of double accessory gallbladder in a living human in 1911 [3].

**Fig. 6:**
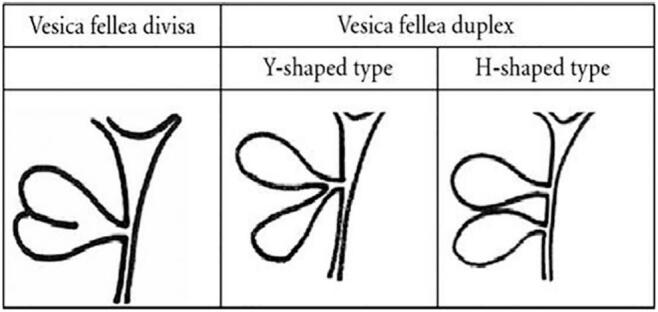
Boyden's classification of gallbladder duplication.

There are two classification systems for duplicated gallbladder. The most widely accepted classification for double gallbladder Is the Boyden's classification [1]. Boyden was the first to describe the duplicate gallbladder and its variable anatomy in 1926. Based on their relation to the cystic duct, he described *“vesica fellea divisa”,* (bilobed gallbladder which is drained by a sole cystic duct), and *“vesica fellea duplex”* (true gallbladder duplication). The latter is sub-classified into the “Y-shaped type” (two cystic ducts uniting before entering the common bile duct), and the “H-shaped or ductular type” (two cystic ducts enter separately into the common bile duct) {Fig. 6} [1]. In our case, there was a duplicated Gallbladder with a single cystic duct and supplied by a single cystic artery (Vesica fella divisa).

The second classification system is Harlaftis classification. It is divided into 2 main groups based upon embryogenesis. Type 1, or split primordial group, is subdivided into septated, V shaped which accounts for 8.5 %, or Y shaped accounting 25.3 %. Therefore, when the cystic primordium splits during embryogenesis, both gallbladders share a common cystic duct. Type 1 septate duplicate gallbladder occurs when there is a single cystic duct and a septum that divides the 2 gallbladders. Type 2 describes accessory gallbladders that are ductular (47.2 %) or trabecular (2.1 %), meaning that they arise from separate primordium from the biliary tree and have individual cystic ducts [6].

There are no specific symptoms or signs associated with duplicate gallbladders. A higher frequency of pathology (malignant or not) in a duplicated gallbladder compared to a single gallbladder has not been confirmed. Patients present atypical symptoms for biliary disease or the common pathologies that can occur as a normal, single gallbladder, including acute cholecystitis, cholelithiasis, empyema, torsion, cholecystocolic fistula, and a mass in the abdomen [7].

Abdominal ultrasound (US) is often the first-line imaging modality used in the assessment of a patient with gallbladder disease; however, it does not always allow a precise diagnosis of gallbladder malformations. Even though it can identify a duplicate gallbladder in the presence of two cystic structures occupying the gallbladder fossa, US is not accurate enough to depict properly the anatomy of the cystic duct(s) and to exclude a wide range of alternative diagnoses [7]. In a review study of 17 case reports, abdominal ultrasound confirmed duplicate gallbladder in only 3 cases [8].

Our patient had three abdominal ultrasounds done at different times by different radiologists for different unrelated diseases requiring an abdominal scan, none of which detected a duplicated gallbladder. Therefore, for patients suspected of having duplicated gallbladder or any other biliary tree abnormality alternative imaging modality should be considered. MRCP has no risk of radiation and is a safe choice [9]. CT cholangiography should be used where there are any contraindications to MRCP or where it is unable to identify adequately the biliary anatomy [7]. Imaging can aid in preoperative suspicion of duplicated gallbladder by revealing two distinct gallbladders with separate cystic ducts connecting to the common bile duct. Anatomical variations may include separate necks, cystic arteries, and independent blood supplies, leading to lobulated or “yin-yan” appearances. The presence of two gallbladder stones is another potential indicator.

Preoperative diagnosis is crucial to plan the surgical approach and proper understanding of biliary tract anatomical variation, aids the surgeons to avoid potential iatrogenic bile duct injuries during cholecystectomy or reoperation in the event that the accessory gallbladder was missed during initial surgery [10].

Surgery is not indicated when duplicated gallbladders are discovered incidentally and prophylactic cholecystectomy in an asymptomatic patient is not recommended but only in symptomatic patients [9]. There has been an emphasis on the need for open cholecystectomy to identify and manage the different types of gallbladder duplication in the literature [11]. In our patient, lacking the facility for laparoscopic cholecystectomy, we proceeded with an open cholecystectomy after accurately identifying the biliary trees anatomy.

On the other hand, with the advent of newer imaging modalities and expertise, these rare anomalies can be diagnosed preoperatively and can be successfully treated by laparoscopy with minimal morbidity [4]. Previously it was recommended that in suspected duplication, open cholecystectomy should be performed to expose the biliary anatomy accurately. However, laparoscopic cholecystectomy has become the procedure of choice if the gallbladder infundibulum-cystic duct junctions are clearly identified. The most commonly performed laparoscopic cholecystectomy is excision of a “V shaped” type gallbladder duplication [12].

The frequency of complications associated with the laparoscopic approach for duplicate gallbladder has not been well studied because of the small number of reported cases. However, the available data showed no increased risk of biliary leak or gallbladder cancer. The risk of conversion rate is slightly higher due to risk of bleeding associated with intrahepatic dissection [7].

## 4 Conclusion

Duplication of the gallbladder is a rare congenital abnormality, which requires special attention to the biliary ductal and arterial anatomy. Although ultrasound is an initial diagnostic modality for suspected gallbladder disease, it can miss the diagnosis of duplicated gallbladder. In the case of incidental intraoperative diagnosis as in our case, it is critical to identify biliary ductal and arterial anatomy to avoid inadvertent injury. Laparoscopic cholecystectomy is the mainstay of treatment in an ideal setup.

## Abbreviation

CT: computed tomography
MRCP: magnetic resonance cholangio-pancreatography

## Patient consent

The report was done with patient's consent.

## Ethics approval

No ethics approval was needed as the case was encountered incidentally and it doesn't involve any human or animal experiment.

## Funding

None.

## Author contribution

Tsion Ketema, MD - Study concept and design, writing the paper, literature review and involved in the management of the patient.

Simeon Mulugeta Mengistu, MD -Involved in acquisition of data and literature review of the paper.

Goytom Knfe Tesfay, MD - Editing and critical review of the paper.

Dagmawi Dagne, MD - Involved in the patient management, acquisition of data and critical review of the paper.

Enku Shiferaw, MD - Involved in the patient management, acquisition of data and critical review of the paper.

## Guarantor

Tsion Ketema, MD.

## Research registration number

IJSCASEREPORTS-D-24-00903.

## Conflict of interest statement

The authors declare that there are no conflicts of interest on this case report.
